# Severe toxicity caused by sorafenib in hepatocellular carcinoma match the data from renal cell carcinoma

**DOI:** 10.1038/bjc.2012.25

**Published:** 2012-01-31

**Authors:** A Lamarca, J Feliu, J Barriuso

**Affiliations:** 1 H U La Paz, Department of Medical Oncology, Castellana 261, 28046, Madrid, Spain

**Sir**,

We read with interest the short communication by Di Fiore *et al* (2011), reporting the correlation between survival and grade 3–4 toxicities in advanced renal cancer patients treated with tyrosine kinase inhibitors (TKIs). As it had been done previously in intestinal stromal digestive, renal, lung and colorectal cancer treated with anti-target drugs, there seems to exist a direct relation between the development of mechanism-based toxicities as rash, hypertension, hypothyroidism and the response to the treatment (Dienstmann *et al*, 2011). The authors evaluated retrospectively 38 patients treated with sunitinib and sorafenib. They evaluated predictive and prognostic factors related to survival, suggesting that grade 3–4 clinical TKI-related toxicities (digestive, cardiac, dermatologic and asthenia) are associated with a significant improvement (grade 1–2 *vs* 3–4 HR 5.55 (CI 95% 1.23–24.9) of overall survival.

In relation to the work presented, we have done a prospective evaluation of 40 advanced hepatocellular carcinoma patients treated with sorafenib between 2002 and 2011 in our centre. In our work, we looked for prognosis and predictive factors of response to sorafenib. We analysed epidemiological factors, previous treatments, comorbidity, performance status and the origin of the hepatic disease. The patients received sorafenib (400 mg twice a day; Llovet *et al*, 2008) and during the treatment, toxicity (CTCAE 4.3, 2010) and response were monitorised. In all, 82% of patients were men, with a medium age of 67 years (range 40–82). In all, 92% of patients were Child A, and 82.5% were BCLC-C. Chronic hepatitis C virus infection was the predominant cause of liver disease (40%), followed by alcohol consumption (20%). The mean number of cycles of sorafenib administrated was 4 (understanding a cycle of treatment as 28 days without discontinuation; range 1–24). According to RECIST criteria v.1.1, the disease control rate was 25% (CI 95% 17–35; 22.5% disease stabilisation and 2.5% partial response), with a progression-free survival of 19.7 weeks (CI 95% 9.8–29.58) and an overall survival of 24.1 weeks (CI 95% 6.09–42.19). These results were similar to those obtained in the SHARP trial (Llovet *et al*, 2008). Nine patients (22%) developed grade 3–4 toxicity: three (7.5%) diarrhoea, two (5%) asthenia and four (10%) hand-foot syndrome. Looking for prognostic factors, our results suggest that there is a significant improvement of progression-free survival (*P*=0.05) for those patients who develop severe skin toxicity.

Clearly, our results are going in the same direction as Di Fiore *et al* (2011). We also agree with the authors that toxicity could be linked to efficacy; however, this has not been shown in early drug development (Postel-Vinay *et al*, 2009). The authors of this later report did not find any correlation between the efficacy and the dose received by patients in targeted agent phase I trials. This later aspect could underline the importance of long-term and cumulative toxicities, which are not well studied in classical phase I trials.

The increasing amount of evidence about the linkage between toxicity and efficacy needs to be tested in randomised trials.
